# Leaf Apoplast of Field-Grown Potato Analyzed by Quantitative Proteomics and Activity-Based Protein Profiling

**DOI:** 10.3390/ijms222112033

**Published:** 2021-11-06

**Authors:** Kibrom B. Abreha, Erik Alexandersson, Svante Resjö, Åsa Lankinen, Daniela Sueldo, Farnusch Kaschani, Markus Kaiser, Renier A. L. van der Hoorn, Fredrik Levander, Erik Andreasson

**Affiliations:** 1 Department of Plant Protection Biology, Swedish University of Agricultural Sciences, SE-234 22 Lomma, Sweden; Erik.Alexandersson@slu.se (E.A.); Svante.Resjo@slu.se (S.R.); Asa.Lankinen@slu.se (Å.L.); Erik.Andreasson@slu.se (E.A.); 2 Plant Chemetics Laboratory, Department of Plant Sciences, University of Oxford, South Parks Road, Oxford OX1 3RB, UK; daniela.sueldo@ntnu.no (D.S.); renier.vanderhoorn@plants.ox.ac.uk (R.A.L.v.d.H.); 3 Chemische Biologie, Zentrum für Medizinische Biotechnologie, Fakultät für Biologie, Universität Duisburg-Essen, Universitätsstr. 2, 45117 Essen, Germany; farnusch.kaschani@uni-due.de (F.K.); markus.kaiser@uni-due.de (M.K.); 4 Department of Immunotechnology, Lund University, SE-221 00 Lund, Sweden; fredrik.levander@immun.lth.se; 5 National Bioinformatics Infrastructure Sweden (NBIS), Science for Life Laboratory, Lund University, SE-221 00 Lund, Sweden

**Keywords:** ABPP, apoplast, proteomics, serine hydrolases, β-glycosidases, potato, field-omics

## Abstract

Multiple biotic and abiotic stresses challenge plants growing in agricultural fields. Most molecular studies have aimed to understand plant responses to challenges under controlled conditions. However, studies on field-grown plants are scarce, limiting application of the findings in agricultural conditions. In this study, we investigated the composition of apoplastic proteomes of potato cultivar Bintje grown under field conditions, i.e., two field sites in June–August across two years and fungicide treated and untreated, using quantitative proteomics, as well as its activity using activity-based protein profiling (ABPP). Samples were clustered and some proteins showed significant intensity and activity differences, based on their field site and sampling time (June–August), indicating differential regulation of certain proteins in response to environmental or developmental factors. Peroxidases, class II chitinases, pectinesterases, and osmotins were among the proteins more abundant later in the growing season (July–August) as compared to early in the season (June). We did not detect significant differences between fungicide Shirlan treated and untreated field samples in two growing seasons. Using ABPP, we showed differential activity of serine hydrolases and β-glycosidases under greenhouse and field conditions and across a growing season. Furthermore, the activity of serine hydrolases and β-glycosidases, including proteins related to biotic stress tolerance, decreased as the season progressed. The generated proteomics data would facilitate further studies aiming at understanding mechanisms of molecular plant physiology in agricultural fields and help applying effective strategies to mitigate biotic and abiotic stresses.

## 1. Introduction

In an agricultural field, plants are continuously exposed to varying climate conditions and challenged by mu below- and above-ground microbes, which can trigger morphological and molecular changes. For example, compared to plants grown under greenhouse conditions, Arabidopsis grown in a field displays a different leaf morphology [1] and apoplastic proteome profile [2] indicating changes in response to the stresses or variable environmental condition. Therefore, molecular studies performed under laboratory-based conditions investigating response to single stress might not translate directly to field conditions where there are multiple confounding stresses [2,3]. Field experiments are an integral component to test applicability of laboratory-based findings, and they are necessary to solve agricultural challenges (for example, in relation to plant breeding and plant protection). Therefore, field experiments are important to gain basic knowledge about the biology of plant field performance and ecology and to understand the differences in physiology and molecular function of plants growing under field conditions. This is crucial to facilitate applicability of field-based studies (for instance, creating a basis for decision support systems in agriculture based on molecular knowledge) [4].

Potatoes (*Solanum* *tuberosum* L.; 2*n* = 4*x* = 48), the third most important food crop in the world, are exposed to multiple biotic and abiotic stresses [5]. The plant apoplast is an important arena in plant–microbe interactions [6,7,8] and plays a crucial role in the plant’s response to abiotic stresses [9], as proteins from the plant and the attacking pathogens are secreted into the apoplast [7,8]. Apoplastic proteomic studies are crucial to understand defense response against pathogenic microbes [10,11]. In potatoes, many proteins in the leaf apoplast change in abundance following the application of host defense inducers [12,13] and *P. infestans* inoculation [14]. An apoplastic study has also been used for predicting activation of defense response in *Solanum* species growing under natural and agricultural conditions [15]. 

Omics techniques have been used in laboratory-based studies to better understand potato response to biotic [14,16,17] and abiotic stresses [18,19,20]. However, application of these techniques to elucidate the molecular processes in field-grown plants, the so called field-omics approach, is scarce [21]. Label-free quantitative proteomics, based on sensitive and reliable mass spectrometry, has emerged as a powerful tool to investigate plant responses to biotic stress and made it possible to investigate differential abundance of proteins in many different systems [14,22,23]. Nevertheless, a change in abundance of a protein identified by quantitative proteomics does not necessarily translate into a change in activity. Recent developments in activity-based protein profiling (ABPP), a method that uses chemical probes that irreversibly bind to the active residue of distinct protein classes in a complex proteome sample, can be used to investigate the functional status of the proteins [24]. This powerful tool has been successfully applied to enhance molecular understanding of different plant–microbe interactions [25,26] and the plant’s response to biotic stresses [27]. ABPP is used to determine the activity of certain protein families, including serine hydrolases and β-glucosidases, in plants challenged by biotic and abiotic stresses [26,27]. Serine hydrolases, carrying an activated serine residue in the catalytic triad, are a large superfamily of enzymes that includes proteases, esterases, lipases, and peptidases [24,26]. β-Glucosidases are glycoside hydrolases that carry a glutamate and aspartate residue at the catalytic site [28]. These enzyme groups are important in cellular processes such as cell wall remodeling, development, biotic and abiotic stress responses. However, to the best of our knowledge, ABPP has not been applied in studies conducted in plants under field conditions.

The objective of this study was to investigate the apoplastic proteome using label-free quantitative proteomics and ABPP to identify apoplastic proteins under field conditions. Therefore, we investigated the apoplastic proteome of field grown potato plants from two different years and followed the change in the abundance of apoplastic proteins during the course of the growing season. Moreover, we performed ABPP on serine hydrolases and β-glucosidases, to understand the functional state of proteins involved in some biological processes.

## 2. Results and Discussion

### 2.1. Analysis of Apoplastic Peptides

To get insights into the proteome of field-grown plants, we collected the leaf apoplastic fluid from fully expanded potato leaves of field-grown plants from two field sites over a two–year period. The apoplast is in many cases the first plant compartment that directly interact with the environment and does not contain any dominating protein such as rubisco. Therefore, we chose to analyze this part of the potato proteins under field conditions. The leaf apoplastic fluid was collected using our mobile laboratory [21,29,30] that allows us to isolate, aliquot, and freeze the samples directly on the site, thus reducing the probability of sample degradation, which can be a problem in proteome sampling [31].

In total, we identified 3960 peptides, of which 501 were identical except for the charge differences of the intact peptide precursor ion detected by MS. Average abundance was computed for the identical peptides resulting in 3459 unique peptides, corresponding to 1257 proteins, which were subjected to quantitative analysis. Of the 3459 peptides, 2335 peptides were uniquely identified for a protein in the sequence database. Therefore, they were designated as diagnostic peptides. The use of our assembled RNA-seq data obtained from potato Desirée (cv.), SW93-1015 (breeding line), and Sarpo Mira (cv.) improved peptide identification by 6% as estimated by the fraction of peptides mapped uniquely to accessions in the RNA-seq data. This increase in peptide identification was however less than the previously reported 17% [14], maybe due to the fact that we used a different potato cultivar Bintje, not represented in our RNA-seq database used for peptide identification. We identified 85 of the 104 peptides previously reported to be have prediction potential for *P. infestans* resistance in leaf and tuber, as well as for tuber yield, in the potato clones SW93-1015 and Desirée [32]. 

**Figure 1:**
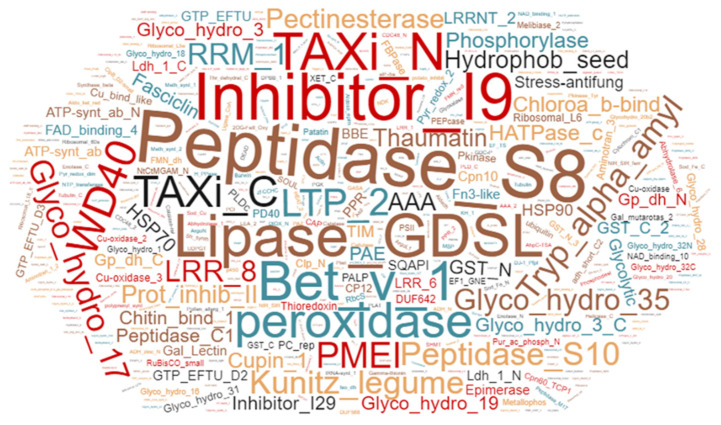
Word-cloud representation of the leaf apoplastic proteome from potato cultivar Bintje. Identified proteins were classified into families using Pfam analysis. Scale of the fonts and colors in the word cloud represents relative abundance of the protein family in the apoplast samples from field-grown plants.

Of the 1257 proteins, 419 were predicted to contain a signal peptide as determined by SignalP version 4.1 [33]. This is in line with the previous studies [6,26], where 30–60% of proteins identified in the apoplast contained a classical signal peptide. Furthermore, Pfam analysis of proteins identified in samples from field-grown plants identified 461 protein families. Peptidases, peroxidases, glycosyl hydrolases, protein GDSL lipases, thaumatin, heat shock proteins, Leucine rich repeat proteins, and epimerases being the predominant protein families in the apoplast of field-grown potato cv. Bintje (Figure 1).

In our dataset, the correlation between peptide abundance and proportion of samples with missing values was *r*^2^ = −0.31 (Supplementary Figure S1). Such a negative correlation between peptide abundance and missing values is expected and has previously been reported [34].

### 2.2. Effect of Fungicide Application on Potato Apoplastic Proteome

In order to investigate whether the application of a fungicide to control a disease change the abundance of proteins in the apoplast, we compared fungicide treated samples with untreated ones. The fungicide application may directly or indirectly, by changing the microbial communities [35], affect the apoplastic proteome of the plants. Some of the field plants were treated with fungicide Shirlan (classified as non-systemic), targeting foliar and tuber late blight infections in potato. According to the Public Release Summary on the fungicide [36], spraying with C^14^-tagged fluazinam (500 g L^−1^), the active ingredient in Shirlan, revealed small traces of the label in the potato pulp, showing some translocation of the fungicide into the plant. We performed separately two-group comparison (*t*-test) analysis in Qlucore for 12 samples from 2011 and 18 samples from 2012 in Mosslunda; however, no differentially abundant protein was detected between fungicide-treated and untreated plants in both years in Mosslunda (*q* < 0.1) (data not shown). Therefore, we conclude that the application of fungicide Shirlan did not significantly change the abundance of the apoplastic proteins in potato leaves. Further studies investigating levels and timing of fungicide application, and its translocation and stability in plant tissues would provide crucial insights into its effects on apoplastic proteome.

### 2.3. Apoplastic Proteome Differences between Growing Sites and between Years

**Figure 2:**
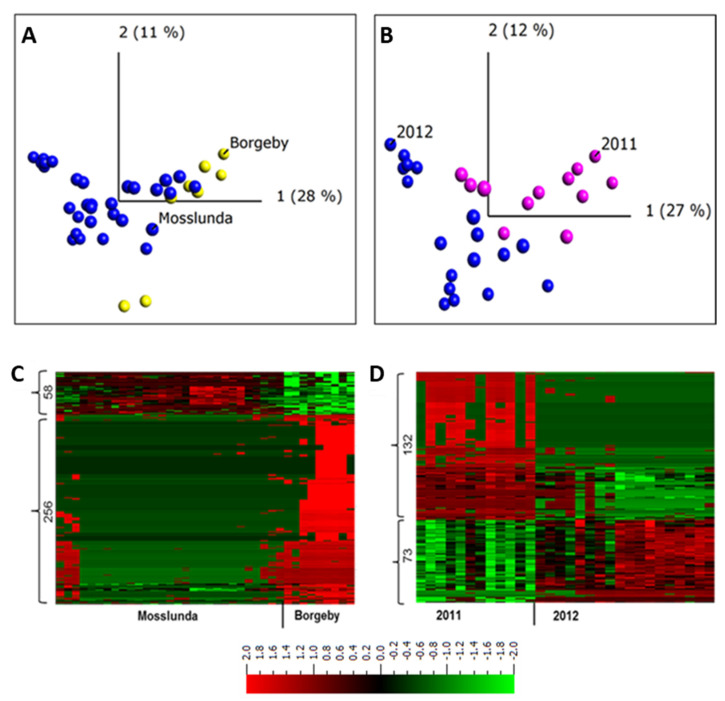
Principal components and heat map analyses of apoplastic proteome samples isolated from potato cultivar Bintje grown at two experimental sites (Borgeby and Mosslunda) and in two different growing seasons (2011 and 2012) in Mosslunda. (**A**) Unsupervised principal component analysis plot of the samples in Mosslunda and Borgeby; (**B**) Unsupervised principal component analysis plot of the samples in Mosslunda in 2011 and 2012; (**C**) Abundance of peptides from Borgeby respectively compared to those in Mosslunda; (**D**) Abundance of peptides collected in 2011 respectively compared to those collected in 2012 in Mosslunda. Two-group comparisons (*t*-test) were performed in Qlucore with false discovery rate using Benjamini−Hochberg correction (*q* < 0.001). Heat maps are sorted using hierarchal clustering and red represents higher abundance (Fold change, log2).

To further understand and describe the dynamics of apoplastic proteome in potato leaves growing in field conditions, we compare the data set between the two sites Borgeby in 2010 and in Mosslunda both in 2011 and 2012 growing seasons. The PCA clustered the samples from the two growing sites together (Figure 2A).

Similarly, ref. [2] found an overlap in apoplastic proteome composition of *Arabidopsis* plants collected from two field sites. It has been shown that the effect of weather conditions on tuber proteome of potato grown at two different fields is minimal [37]. The overlap between field samples observed in the PCA plot indicates the similarity between the apoplastic protein compositions of the potato plants grown in Mosslunda and those grown in Borgeby (Figure 2A). This indicates similar apoplastic proteome profile among the plants grown in these sites. However, a transcriptome analysis of grapevine berries grown in different sites identified more than 8000 differentially expressed genes [38]. 

To describe individual proteins that were differentially regulated between potato plants grown at these two sites, we performed a two-group comparison (*t*-test) in Qlucore (*q* < 0.001) and found 314 peptides (9%) from 234 proteins (Figure 2C). These results indicate that, despite the overlap of Mosslunda and Borgeby samples in the PCA plot, hence similarity in apoplastic proteome, not all of the proteins had a similar pattern of abundance at both growing sites. The abundance of subtilisin-like proteases (Q9LWA4), endochitinase (DMP400046624), Kunitz trypsin inhibitor (DMP400046980), pectinesterase (DMP400055021), and peroxidases (DMP400052953, Q9SD46) were higher in Borgeby plants compared to those grown in Mosslunda and those of glucan endo-1,3-β-D-glucosidase (DMP400051976), glyceraldehyde-3-phosphate dehydrogenase (DMP400017652), and methionine synthase (DMP400048869) were lower (Supplementary Table S2).

Similarly, to investigate the differences between the years, apoplastic samples were collected in 2011 and 2012 from plants grown in Mosslunda. The PCA analysis grouped the samples from the same year together, but there was an overlap between the samples from both years (Figure 2B). A two-group comparison (*t*-test) analysis in Qlucore (*q* ≤ 0.001) found 205 peptides (6% of total peptides, corresponding to 156 proteins) differentially abundant in 2011 and 2012 in Mosslunda (Figure 2D). This indicates that relatively few apoplastic proteins differ in abundance between the years. Compared to in 2012, osmotin-like protein (Q41350), pectinesterase (DMP400031280), PAE (DMP400041742), polygalacturonase (DMP400021809), and β-galactosidase (DMP400026688) showed increased abundance in 2011 (Supplementary Table S3). Dal Santo et al. [38] found 625 grapevine genes differentially expressed in at least one of the three growing seasons. Although investigation across more years and locations is required to draw solid conclusions, the result indicates stability of apoplastic proteome profile in potato. It is also possible that, given the sites are in the same climate conditions; the variation in weather and biotic condition was minimal thus did not significantly alter the apoplastic proteome.

### 2.4. Abundance of Apoplastic Proteins across a Growing Season

**Figure 3:**
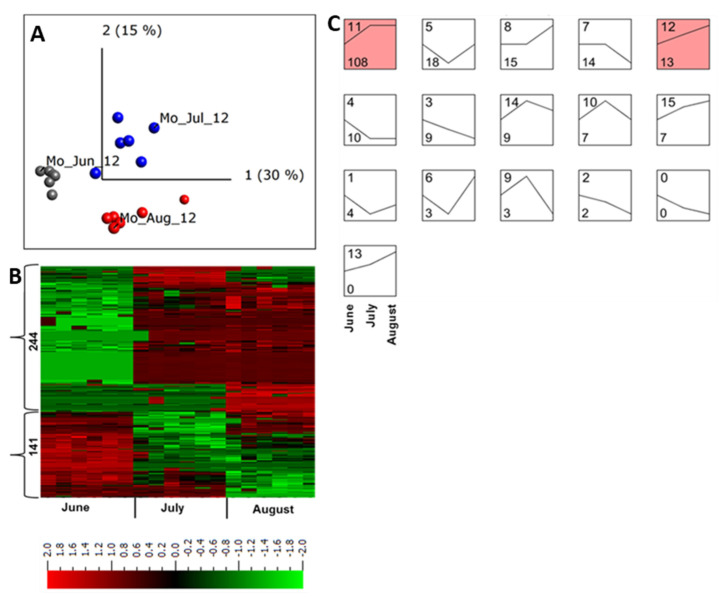
Quantitative analysis of apoplastic proteome samples isolated from potato cultivar Bintje grown in Mosslunda in 2012. (**A**) Unsupervised principal component analysis plot of all the samples collected in June (Mo_Jun_12), July (Mo_Jul_12), and August (Mo_Jul_12). Each circle represents one biological replicate. (**B**) Heat maps and the number of peptides up- and down-regulated in plants grown under field conditions in Mosslunda. We performed a multi-group comparison with false discovery rate < 0.001 (according to the Benjamini−Hochberg procedure for determining *q*). Heat map of the differentially regulated peptides (*q* < 0.001) was sorted using hierarchal clustering and red represents higher abundance (Fold change, log2). (**C**) STEM clustering analysis of apoplastic peptides in June, July, August of 2012 in Mosslunda. Proteins that were significantly (*q* ≤ 0.001) increased or decreased in at least one of the months across the growing season were used for the STEM clustering analysis. Top left of each box is the profile number and bottom left of each box indicates the number of peptides that fit the defined abundance pattern in June, July, and August. The STEM analysis identified 16 profiles, of which profiles 11 and 12 contains statistically significant number of proteins (*p* < 0.05).

Under field conditions, microbial populations and abiotic stresses change throughout the growing season [39,40] which might accordingly alter the apoplastic proteome profile. To understand the possible changes in the apoplastic proteome within the same growing season, plant samples without disease symptoms collected in June, July, and August in Mosslunda were investigated. The samples from the same month were clustered together, and a multi-group comparison (*q* ≤ 0.001) identified 320 peptides (9.3%) from 240 proteins that were differentially regulated in samples collected in at least one of these months (Figure 3A,B).

STEM clustering [41] of differentially regulated proteins identified 16 abundance pattern profiles for proteins that were co-regulated throughout the growing season (Figure 3C). Of those, only two were significant profiles (*p* < 0.05). Profile 11 represented proteins with lower abundance in June and their abundance increased at a similar degree in July and August, whereas profile 13 comprised proteins with continued increase in abundance during the season (Figure 3C).

In July and August most of the proteins with increased abundance in profile 11 were involved in plant response to specific biotic and abiotic stresses (Table 1, Figure 3C, profile 11).

Proteins with increased abundance in July and August included peroxidases (DMP400022299, DMP400055305), serine carboxypeptidase III (DMP400000884), class II chitinase (DMP400002757), pectinesterases (DMP400016183, Q43143), and osmotins (DMP400005465, DMP400005463, Q5XUH0). In addition, ceramidase (DMP400007784), 1,3-β-glucan glucanohydrolase (Q70BW9), glucan endo-1,3-β-D-glucosidase (DMP400051976), and Kunitz trypsin inhibitor (DMP400046980) also showed increased abundance (log_2_ fold change ≥ 4) in July and August compared to that in June (Table 1; Figure 3C, profile 11). This was corroborated by our finding that the number of potato plants with activated immunity increased (as measured as PR protein accumulation) at the end of the growing season, which might be associated with increased presence of pathogens as the season progressed [15].

### 2.5. Difference in Protein Abundance under Field and Greenhouse Conditions

**Figure 4:**
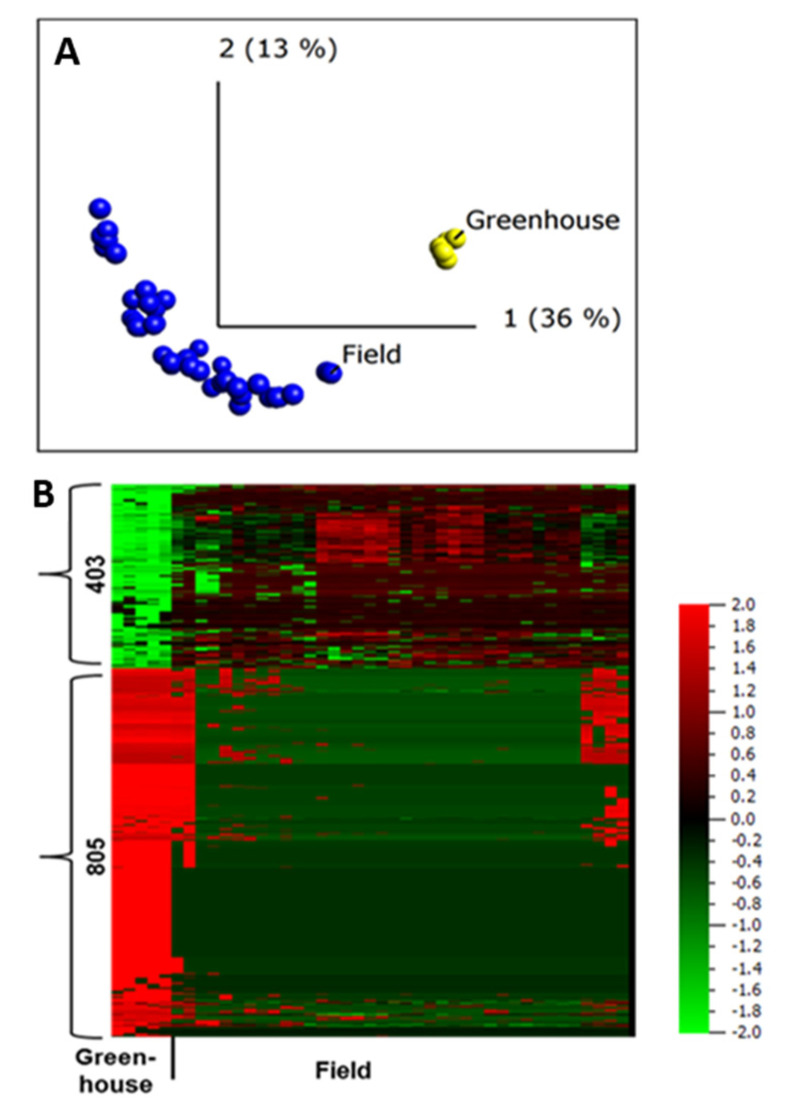
Quantitative analysis of apoplastic proteome samples isolated from potato cultivar Bintje grown under greenhouse and field conditions. (**A**) Unsupervised principal component analysis plot of all the samples. Each circle represents one biological replicate; (**B**) heat maps and numbers of peptides up- and down-regulated under greenhouse and field conditions according to a two-group comparison in Qlucore with a false discovery rate < 0.001 (according to the Benjamini−Hochberg procedure for determining q). The heat map of the differentially regulated peptides (*q* < 0.001) was sorted using hierarchal clustering and red represents higher abundance (Fold change, log2).

To investigate the apoplastic proteome differences between plants grown under greenhouse and those grown under field conditions, we first conducted an unsupervised PCA. The resulting PCA plot showed a clear clustering of the field- and greenhouse-grown samples into different groups (Figure 4A), regardless of the sampling year and growing site, suggesting distinct apoplastic proteome profiles for field- and greenhouse-grown plants.

This is in agreement with a study that compared apoplastic proteome profiles between field- and laboratory-grown *Arabidopsis* using a limited number of samples [2]. Our data also strengthens the notion that peptide biomarkers developed using apoplastic proteome of field-grown potato can be a powerful tool for trait prediction [32].

A two-group comparison (*t*-test) in Qlucore, field vs. greenhouse, identified 1208 peptides that belong to 606 proteins, making up to 48% of all the proteins identified in the apoplast, to be differentially abundant in plants grown in the field and greenhouse (*q* ≤ 0.001). Of those, 781 peptides were diagnostic, assigned to only one protein in our database. The abundance of 805 peptides was lower in field-grown samples compared to those grown in the greenhouse (Figure 4B). This difference in abundance of most of the proteins in greenhouse samples might be due to differences in extraction efficiency associated with variation in leaf anatomy between plants grown in greenhouse and field conditions [1], or a limited sampling of the greenhouse samples.

To describe the identified proteins that were differentially abundant under field and greenhouse conditions, we carried out a MapMan analysis [42] using 350 differentially abundant proteins with PGSC identity numbers (Supplementary Figure S2). The analysis identified many stress-related proteins such as proteins associated with proteolysis, proteins involved in cell wall synthesis or degradation, proteins classified as pathogenesis-related proteins (PR-proteins), peroxidases, and proteins involved in signaling; most of these proteins were at lower abundance in field-grown plants than in plants grown in the greenhouse (Supplementary Figure S2A). However, most of the redox and heat shock proteins were more abundant in field-grown plants (Supplementary Figure S2B).

### 2.6. ABPP Reveals Seasonal Effects

**Figure 5:**
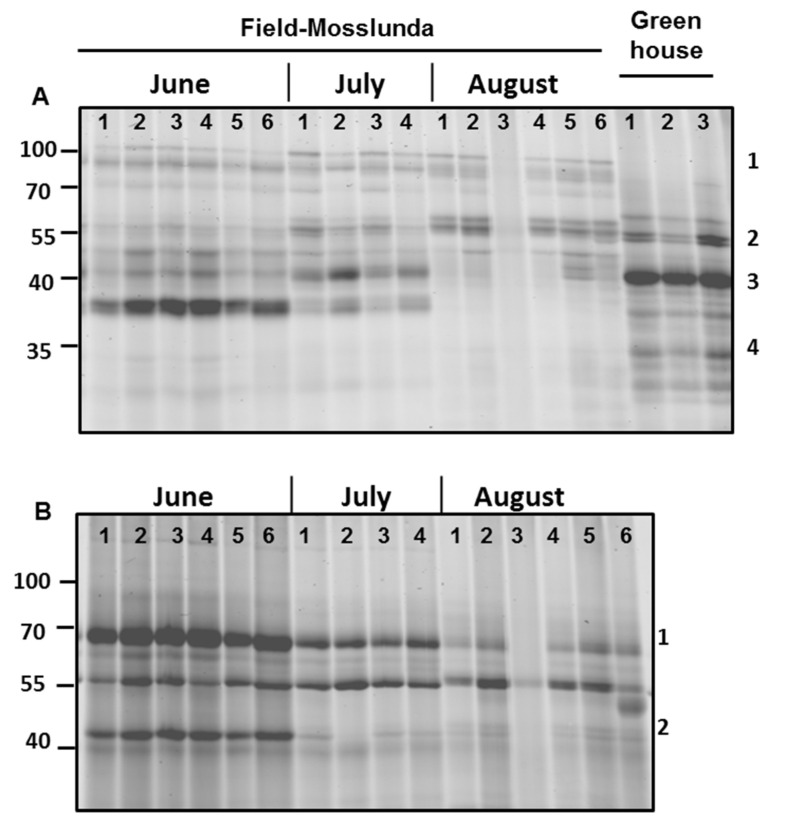
Serine hydrolase and β-glycosidase activity profiling of potato cultivar Bintje grown under greenhouse and field conditions in Mosslunda in June, July, and August 2012. Apoplastic proteins were labeled by 2 µM probe for (**A**) serine hydrolase and (**B**) β-glycosidase. The probe-labelled proteins were separated on 12% sodium dodecyl sulfate-polyacrylamide electrophoresis gels and detected using a fluorescence scanner.

Serine and glycosyl hydrolase protein families are commonly found in the apoplast [6]; both families were among the most abundant in our samples (Figure 1). Similarly, we also found that most of the differentially regulated proteins in field-grown plants vs. greenhouse-grown plants were associated with catalytic and hydrolytic activities, as shown using MapMan pathway analysis (Figure 5, Supplementary Figure S2). Therefore, we studied the activity profile of serine hydrolase and β-glucosidase proteins in the apoplast using ABPP. The labeling of the apoplast for active serine hydrolases identified a signal (#1) at 100 kDa only in the field-grown potato plants (Figure 5A). In contrast, a signal (#3) at 40 kDa had higher intensity in greenhouse- than in field-grown plants. 

Surprisingly, we found that the activity of serine hydrolases and β-glycosidases was generally decreasing as the growing season progressed (Figure 5). The intensity of the signal (#4) below 40 kDa for active serine hydrolases decreased from June to August in field-grown plants (Figure 5A). Using a probe for β-glycosidases, we identified that intensity signal (#1) at 70 kDa and signal (#2) at 40 kDa decreased later in the season (Figure 5B), showing decreasing activity of these proteins as the season progressed.

### 2.7. Serine Hydrolases and β-Glycosidases Identified by ABPP and MS

**Figure 6:**
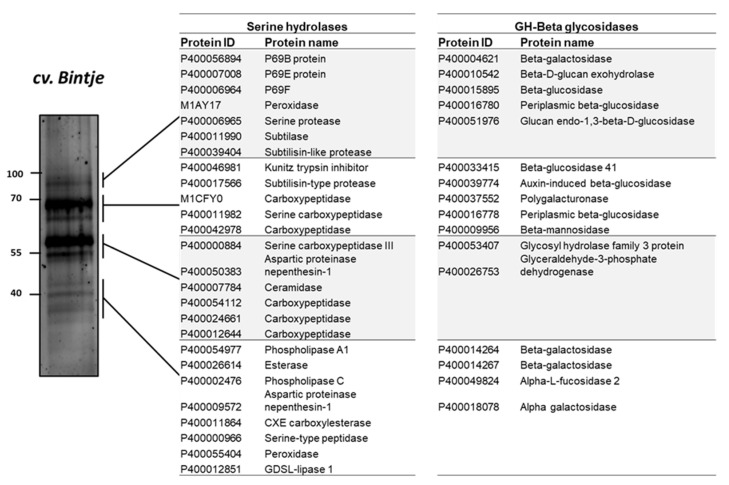
Identification of serine hydrolases and β-glycosidases proteins that were captured by activity-based probes. Leaf apoplastic proteome of the potato sample was co-labelled by 5 μM biotinylated probes for β-glucosidase (JJB111) and serine hydrolases (FP-biotin). Biotinylated proteins were then affinity-purified with streptavidin beads and separated on 12% sodium dodecyl sulfate-polyacrylamide electrophoresis gel. The gel was stained by SYPRO Ruby staining.

ABPP has been applied in a limited number of protein families (van der Hoorn et al., 2011). In this study, to investigate the proteins identified in the activity profile of the potato apoplastic fluid, the samples were purified after labelling with a mix of two biotinylated probes targeting serine hydrolases and β-glycosidases. The detected protein bands in ABPP were excised and analyzed using MS. The results of the MS analysis of the apoplastic proteome, which was used to identify the composition of protein signals, revealed that serine hydrolase signal (#1) at 100 kDa corresponded to subtilisin-like proteins, such as P69B (DMP400056894), P69E (DMP400007008), P69F (DMP400006964), subtilase (DMP400011990), and serine protease (DMP400006965) (Figure 6).

Proteins corresponding to signals #3 and #4 included carboxylesterase (DMP400011864), esterase (DMP400026614), serine-type peptidase (DMP400000966), and GDSL-lipase 1 (DMP4000-12851) (Figure 6); GDSL-lipase 1 is involved in plant defense against pathogens such as *Pseudomonas syringae* [43]. Moreover, β-glycosidases were identified at 100 kDa (β-galactosidases [DMP400004621, DMP4000-15895, and DMP400016780]) and at 70 kDa (polygalacturonase [DMP400037552], β-glucosidase [DMP400033415], β-mannosidase [DMP400009956]), and β-galactosidase (DMP400014264 and DMP400014267) and α-galactosidase (DMP400018078) were detected at 40 kDa (signal #4) (Figure 6). 

## 3. Materials and Methods

### 3.1. Plant Material and Field Sites

Potato cultivar Bintje was used in this study, and field experiments were conducted at two experimental sites in southern Sweden: Mosslunda (55°58′ N, 14°6.3′ E) in 2011 and 2012 and Borgeby in 2010 (55°45′ N, 13°23′ E). The experiment site in Borgeby had a sandy clay soil with 2.8 % humus, 16 % clay content, and 55% fine sand, with pH 7.1. The nutrient concent were Phosphorus (9.9 mg), Potassium (9.9 mg), Magnesium (10 mg), and Calcium (310 mg) per 100 g soil. In Mosslunda in 2011, the soil was sandy (79%), with a low clay content (7%) and humus (4.2%), and the chemical property was pH was 7.2, and Phosphorus (31 mg), Potassium (12 mg), Magnesium (13 mg), Calcium (490 mg) per 100 g of soil. In 2012 in Mosslunda, the soil was sandy (84%), with a low clay content (3%) and humus (4.1%), the pH was 6.5, and Phosphorus (21 mg/100 g), Potassium (11 mg), Magnesium (10 mg), Calcium (200 mg) per 100 g of soil. The average temperature, relative humidity and precipitation during this study was in Borgeby (17.6 °C, 73.8% RH, and 357 mm), and in Mosslunda in 2011 (16.9 °C, 79.9% RH, and 293 mm) and in 2012 (16.0 °C, 78,0% RH, and 213 mm). Furthermore, the monthly weather condition in Mosslunda in 2012 was in June (13.0 °C, 76 % RH, and 190 mm), July (17.1, 79% RH, and 242 mm) and August (17.0 °C, 80.0% RH, and 265 mm). Plants were grown following good experimental practice in accordance with EU directive 93/71, KIFS 2004:4, STAFS 2001:1, and standard operative procedures, SLU 2004 [44]. Plants in the greenhouse were grown at SLU Alnarp (55°65′ N, 13°07′ E), under conditions described previously [44]. Some plants were treated with a fungicide Shirlan (ISK Biosciences Europe S.A., Machelen, Belgium; active ingredient: fluazinam 500 g L^−1^) according to manufacturer’s recommendation (0.3 L ha^−1^). No pathogen-related symptoms were visible on any of the analyzed leaves.

### 3.2. Apoplast Isolation and Protein Digestion

Apoplastic fluid was isolated, between 10 a.m. and 3 p.m., from five fully expanded leaves of a single plant by vacuum infiltration-centrifugation using a mobile field laboratory as previously described [21,29,30]. Isolated samples were aliquoted on site, frozen in liquid nitrogen, and kept at −80 °C until used for either label-free quantitative proteomics analysis or activity-based protein profiling (ABPP). For quantitative proteomics analysis, a 30 µL aliquot of each apoplastic sample was cleaned and trypsin-digested as described in [32] before analysis by mass spectrometry.

### 3.3. Mass Spectrometry

For high-performance liquid chromatography-tandem mass spectrometry (HPLC-MS/MS) analysis, 5 µL of the peptide solution was injected into Eksigent nanoLC-2D HPLC system coupled to a LTQ Orbitrap XL ETD (Eksigent Technologies, Dublin, CA, USA). Peptides were separated using a linear gradient for 90 min at a flow rate of 300 nL min^−1^. The eluted peptide spectra were acquired, analyzed, and the four most intense ions were selected for fragmentation in linear trap quadrupole (LTQ) [45]. The raw data were converted to mzML [46] and Mascot generic format (MGF) files using ProteoWizard [47] and uploaded to the Proteios software environment (ProSE) [48], where the MGF and mzML files were used for MS/MS identification and feature detection, respectively. Protein identification was performed in Mascot (www.matrixscience.com) (accessed on 1 November 2021) and X!Tandem (www.thegpm.org/tandem) (accessed on 1 November 2021) by searching a protein sequence database containing all *Solanum* proteins in Uniprot (http://www.uniprot.org/) (accessed on 1 November 2021) and potato genome project (https://solgenomics.net/) (accessed on 17 June 2015). Protein sequences based on de novo assembled transcripts of potato clones Desirée, Sarpo Mira, and SW93-1015 were included in the database [14]. Reversed sequences of all the proteins in the database (449,968 protein entries) were included as decoys. For MS/MS searches, MS mass tolerance was set to 5 ppm and MS/MS fragment tolerance to 0.5 Da. One missed cleavage, fixed cysteine carbamidomethylation, and variable oxidation of methionine were allowed. All search results at the peptide spectrum level were subsequently filtered at a false discovery rate (FDR) of 1% [48]. Feature detection to quantify peptides was performed on the mzML files from Proteios using Dinosaur [49]. An alignment algorithm was used in Proteios to propagate peptide identities between LC-MS/MS runs [50] and the report was exported for further analysis. After feature matching across all runs with a recall of 0.99 and a precision of 0.80 based on common identifications, the peptide feature level FDR could be estimated to 4% from the fraction of decoy identifications. The proteomics data used for quantitative analysis have been deposited to the ProteomeXchange Consortium (http://proteomecentral.proteomexchange.org) (accessed on 1 November 2021) via the PRIDE partner repository with the dataset identifier PXD006392.

### 3.4. Quantitative Analysis of Peptides

The quantitative dataset with peptide precursor intensities was analyzed using Normalyzer v1.1.1 [51], and after comparing 12 normalization methods the Loess-G was used for normalization [52]. The log_2_-transformed normalized data were used for visualization and statistical tests for identification of differentially abundant proteins; missing values were treated as described in [12]. Qlucore Omics Explorer v3.2 software (http://www.qlucore.se/) (accessed on 1 November 2021) was used to generate principal components analysis (PCA) plots and perform comparative analysis. Unsupervised PCA plots were generated to show similarities or differences between the samples. To identify peptide and proteins with differential abundance, comparative analyses were performed two group comparison (*t*-test) in Qlucore with the Benjamini−Hochberg false discovery ratio (FDR) procedure (*q* ≤ 0.001). Heat maps sorted with hierarchical clustering were generated and the list of peptide and proteins differentially regulated among the samples was exported for bioinformatics analysis. If the protein was represented by two or more peptides, a median abundance value was calculated.

### 3.5. Labelling of Apoplastic Proteome Activity

For the activity-based profiling, 5 µL of 500 mM sodium acetate (NaAc, pH = 5) and 1 µL of 250 mM dithiothreitol (DTT) were added to 43 µL of ice-thawed apoplastic fluid. Two micromoles of each probe for serine hydrolases (FP-Rh) [53] and Glycosyl hydrolases (JJB70) [28] were added to the labeling reaction. For the inhibition tests, equal amounts of labeling reactions were pre-incubated for 30 min at room temperature (RT) with 100 µM of inhibitor 3,4 dichloroisocoumarin (DCI) against each probe. In samples without the inhibitor, DMSO was added instead and all labeling reactions were incubated for 1 h in darkness at RT. The reactions were stopped by adding 15 µL of 4X gel loading buffer, and the whole samples were boiled at 95 °C for 5 min. Proteins were separated on 12% sodium dodecyl sulfate-polyacrylamide electrophoresis (SDS-PAGE) gels and probe-labelled proteins were detected using a Typhoon 9400 fluorescence scanner (GE Healthcare, Bio-Sciences AB, Uppsala, Sweden).

### 3.6. Affinity Purification and Identification of Serine Hydrolases and β-glycosidases

Apoplastic fluid of Bintje (2.5 mL) was incubated with 5 μM biotinylated probes for β-glycosidase (JJB111) [28] and serine hydrolases (FP-biotin) [53], 50 mM NaAc, and 1 mM DTT for 1 h in the dark at RT. The labelled proteins were affinity-purified as described previously [26,28], eluted from streptavidin beads by adding 30 µL of 4X gel loading buffer and boiling at 95 °C for 10 min. The samples were centrifuged and 15 µL of the supernatant was loaded and separated on 12% SDS-PAGE gel. The gel was incubated with SYPRO fix for 30 min and stained overnight in the dark with SYPRO Ruby Protein Gel Stain (Thermo Fischer Scientific, Waltham, MA, USA). Protein bands were detected and excised from the gel using scalpel, and gel pieces were subjected to in-gel tryptic digestion [26,28].

Peptide and protein identification was performed using LC-MS/MS of the in-gel digests on an Orbitrap Elite instrument (Thermo, [54]) that was coupled to an EASY-nLC 1000 liquid chromatography (LC) system (Thermo). The LC was operated in the one-column mode. The analytical column was a fused silica capillary (75 µm × 36 cm) with an integrated PicoFrit emitter (New Objective) packed in-house with Reprosil-Pur 120 C18-AQ 1.9 µm resin (Dr. Maisch). The analytical column was encased by a column oven (Sonation) attached to a nanospray flex ion source (Thermo). The column oven temperature was adjusted to 45 °C during data acquisition. The LC was equipped with two mobile phases: solvent A (0.1% formic acid, FA, in water) and solvent B (0.1% FA in acetonitrile, ACN). All solvents were of UPLC grade (Sigma-Aldrich, St. Louis, MO, USA). Peptides were directly loaded onto the analytical column with a maximum flow rate that would not exceed the set pressure limit of 980 bar (usually around 0.4–0.6 µL/min). Peptides were subsequently separated on the analytical column by running a 50 min gradient of solvent A and solvent B (start with 7% B; gradient 7 to 35% B for 40 min; gradient 35 to 100% B for 5 min and 100% B for 5 min) at a flow rate of 300 nl/min. The mass spectrometer was operated using Xcalibur software version 2.2 SP1.48. The mass spectrometer was set in the positive ion mode. Precursor ion scanning was performed in the Orbitrap analyzer (FTMS; Fourier Transform Mass Spectrometry) in the scan range of *m*/*z* 300–1500 and at a resolution of 60,000 with the internal lock mass option turned on (lock mass was 445.120025 *m*/*z*, polysiloxane) [55]. Product ion spectra were recorded in a data dependent fashion in the ion trap (ITMS) in a variable scan range and at a rapid scan rate. The ionization potential (spray voltage) was set to 1.8 kV. Peptides were analyzed using a repeating cycle consisting of a full precursor ion scan (1.0 × 10^6^ ions or 50 ms) followed by 12 product ion scans (1.0 × 10^4^ ions or 100 ms) where peptides are isolated based on their intensity in the full survey scan (threshold of 500 counts) for tandem mass spectrum (MS2) generation. The MS2 permits peptide sequencing and identification. Collision induced dissociation (CID) energy was set to 35% for the generation of MS2 spectra. During MS2 data acquisition dynamic ion exclusion was set to 60 s with a maximum list of excluded ions consisting of 500 members and a repeat count of one. Ion injection time prediction, preview mode for the FTMS, monoisotopic precursor selection and charge state screening were enabled. Only charge states higher than 1 were considered for fragmentation.

Peptide and Protein Identification was performed using MaxQuant. RAW spectra were submitted to an Andromeda [56] search in MaxQuant version 1.5.3.30 using the default settings. [57] Label-free quantification was activated. MS/MS spectra data were searched against the Uniprot *S. tuberosum* (UP000011115_4113.fasta; 53104 entries) [58], as well as assembled RNA-seq datasets of *Solanum dulcamara* (DUL.fasta; 26392 entries) and *Solanum tuberosum* cv. Desiree (DES.fasta; 24703 entries) [14,16]. Further analysis and filtering of the results was carried out in Perseus version 1.5.5.3 [59]. Proteins unique or with higher spectral count in a specific protein band in the SDS-PAGE are reported herein. The identified proteins with their spectral counts are listed in Supplementary Table S1.

### 3.7. Bioinformatics Analysis

We used Pfam enrichment analysis [60] for investigation of protein families in the apoplastic proteome. SignalP 4.1 was used to predict the presence of secretion signals in identified proteins [33]. In order to identify proteins with a similar differential regulation pattern, we performed STEM structure analysis in STEM v1.3.8 with default parameters [41]. Location of the identified proteins in potato genome was predicted using SPUD DB Genome browser version 1.70 (http://solanaceae.plantbiology.msu.edu/) (accessed on 1 November 2021). Functional categories of identified proteins were determined by gene ontology (GO) enrichment analysis in agriGO version 2.0 [61]. MapMan version 3.6.0 [42] was used for pathway analysis based on the potato mapping file obtained from GoMapMan [62]. To establish a correlation between the quantitative profiling and ABPP, proteins and peptides found by both approaches were identified based on the sequence similarity analysis. The Potato Genome Sequencing Consortium (PGSC) protein numbers throughout this report have been abbreviated for readability; for example, protein PGSCDMP400044750 is abbreviated to DMP400044750. 

## 4. Conclusions

Proteomics can be useful for understanding the physiology of field-grown plants, and we found similarities between years and sites. We show that fungicide may not affect the apoplastic proteome significantly, but this requires a more detailed investigation on the translocation and stability of the fungicide applied and changes in phyllosphere microbial community as well as induction of plant defense responses. Variation in proteomics profile including presence of proteins with differential abundance and activity among samples grown in different sites, growing season, as well as across the months within the growing season indicates dynamic regulation of parts of the apoplastic proteome in response to biotic and abiotic factors. Indeed, most of the differentially regulated proteins were associated with stress related processes. Nevertheless, this warrants further investigation to identify proteins that can be useful for molecular-assisted decision making in management strategies of these stresses. The study combines quantitative analysis with ABPP to gain insights into the actual activity of certain protein classes. This study also shows the importance of collecting apoplastic proteomes in field conditions and that understanding the proteome in agricultural fields would be a new dimension in order to understand the physiological state of field-grown plants (field-omics) and support biotic and abiotic stress mitigation strategies.

## Figures and Tables

**Table 1:** Differentially abundant proteins in plants collected in June, July, or August in 2012 in fields in Mosslunda at false discovery rate < 0.001 (according to Benjamini−Hochberg), corresponding to the abundance pattern identified in STEM clustering profile 11 (Figure 3). Only unique peptides were used for the analysis. Shown are peptides with log_2_ fold change ≥ 4 in July and August compared to their abundance in June.

Peptide Sequence	Protein IDs	Protein Name	Genome	Signal P	Log2 Fold Change	
			Location		July	August
TDPNQNTGIVIQK	DMP400016183	Pectinesterase	chr03	Yes	4.72	4.73
DGQPSEQHFGLFYPDQR	Q70BW9	1,3-beta-glucan glucanohydrolase			4.71	4.81
GQTWVIDAPR	DMP400005465	Osmotin	chr08	Yes	4.68	4.83
GLTWSVPTGR	DMP400022299	Peroxidase	chr01	Yes	4.68	4.63
RLDPGQTWVIDAPR	Q5XUH0	Osmotin-like protein			4.65	4.84
MLNEGFVPDDVSLK	Q9FHR3	Putative pentatricopeptide repeat-containing protein At5g37570			4.65	4.56
NIQNAISGAGLGNQIK	DMP400051976	Glucan endo-1,3-beta-D-glucosidase	chr10	Yes	4.64	4.64
TSNLYAIGEMEIEENKK	DMP400023312	DUF26 domain-containing protein 2	chr12	Yes	4.62	4.67
LLALSDTPYK	DMP400046980	Kunitz trypsin inhibitor	chr06	Yes	4.62	4.57
VCWPVPNK	DMP400033260	Xylem serine proteinase 1	chr10	No	4.61	4.65
SPSAYLNNPAGER	DMP400007784	Ceramidase	chr03	Yes	4.61	4.24
RYCGMLNVPTGEN- LDCNNQR	DMP400002757	Class II chitinase	chr02	Yes	4.6	4.72
QRCPDAYSYPQDD- PTSTFTCPSDSTNYR	DMP400005463	Osmotin OSML13	chr08	Yes	4.59	4.34
GVIFFGDSPYVFLPGMDVSK	DMP400015799	Xyloglucan-specific endoglucanase inhibitor 4	chr01	Yes	4.58	4.49
IFESCSTDTFQIR	DMP400041178	Embryo-specific 3	chr01	Yes	4.57	4.51
YCGICCEECK	DMP400037307	Snakin-1	chr04	Yes	4.57	4.45
ALPTYTPESPADATR	DMP400038185	Transketolase, chloroplastic	chr10	No	4.56	4.62
VITSSTEAQAYTPGR	Q43143	Pectinesterase/pectinesterase inhibitor U1			4.53	4.54
GFEAAPSVSFTVDGEEK	DMP400000884	Serine carboxypeptidase III	chr11	No	4.52	4.62
FVVVVDDSK	M1BPR5	Uncharacterized protein (Solanum tuberosum)			4.52	4.58
AETWVQEETRALISLR	Q43326	Box II Factor			4.52	4.56
KFGLTVDNVLDAR	DMP400031346	Reticuline oxidase	chr02	Yes	4.52	4.52
LCPQGGDGGTFANLDK	DMP400055305	Peroxidase	chr01	Yes	4.51	4.61
CLCGSPLPDCK	DMP400038422	Polygalacturonase inhibitor protein	chr07	Yes	4.51	4.49
TVTNLGDGQSTYTAK	DMP400027005	Subtilisin-like protease preproenzyme	chr12	Yes	4.48	4.51
LCGEIPKGEYMK	DMP400014905	Polygalacturonase inhibiting protein	chr09	Yes	4.45	4.17
ADNLDTCYR	DMP400025990	41 kD chloroplast nucleoid DNA binding protein (CND41)	chr08	Yes	4.43	4.23
GTGDFTGR	SW_g323.t1	Pathogenesis-related protein 1b (Solanum tuberosum)			4.41	4.49
RIVDIPAGAFSFNSNT- GAGTIIDSGTVFTR	DMP400009572	Aspartic proteinase nepenthesin-1	chr01	Yes	4.38	4.55
VIIADIQNDLGNSLVK	DMP400032777	Short chain alcohol dehydrogenase	chr12	No	4.37	4.56
TLPESTTNENK	K7WVA0	Acyl-CoA-binding protein (Solanum tuberosum)			4.37	4.42
CHAVQCTANINGECPGQLK	DMP400023388	Osmotin		Yes	4.35	4.68
TNCNFDGDGR	Q01591	Osmotin-like protein TPM-1			4.35	4.41
LSEDGQVLEVLEDVEGK	DMP400030201	Strictosidine synthase	chr07	Yes	4.31	4.59
SMVGTPLMPGISVDTYIF- ALYDEDLKPGPGSER	DMP400001406	Glucan endo-1,3-beta-glucosidase	chr01	Yes	4.3	4.65
GNLDIFSGR	DMP400035839	Wound/stress protein	chr04	Yes	4.27	4.6
ITGNDYSSGVR	DMP400007118	Citrate binding protein	chr11	Yes	4.26	4.54
AVGEAGLGNDIK	DMP400062364	Glucan endo-1,3-beta-glucosidase, basic isoform 2	chr01	No	4.24	4.58
HAGPQFDYLEK	DMP400019521	Glutathione S-transferase omega	chr10	No	4.23	4.58
SSSTDVFGR	DMP400043338	Subtilisin-like protease	chr02	Yes	4.21	4.61
YLVTIGGVEGNPGR	DMP400017956	Miraculin	chr03	Yes	4.21	4.52
MYQLSFK	DMP400050666	Unidentified	chr08	Yes	4.21	4.48
ADAGHVLVEK	DMP400022826	MRNA binding protein	chr09	No	4.15	4.49
GQGTVGTEINR	DMP400023006	Threonine dehydratase biosynthetic, chloroplastic	chr09	No	4.14	4.52
WQPSGADQAANR	P52405	Endochitinase 3			4.1	4.45

## Data Availability

The data presented in this study are openly available in ProteomeXchange Consortium at [http://proteomecentral.proteomexchange.org] (accessed on 1 November 2021) with Project DOI: [10.6019/PXD006392] and reference number [PXD006392].

## Supplementary Materials

The following are available online at https://www.mdpi.com/article/10.3390/ijms222112033/s1.
