# Supplementary material for: Leaf Apoplast of Field-Grown Potato Analyzed by Quantitative Proteomics and Activity-Based Protein Profiling
Source: Int J Mol Sci. 2021 Nov 6;22(21):12033. doi: 10.3390/ijms222112033 (PMC8584485; doi:10.3390/ijms222112033)
Supplement: Supplementary file 1 [file ijms-22-12033-s001.zip › Supplementary Figures.pdf]

Supplementary materials

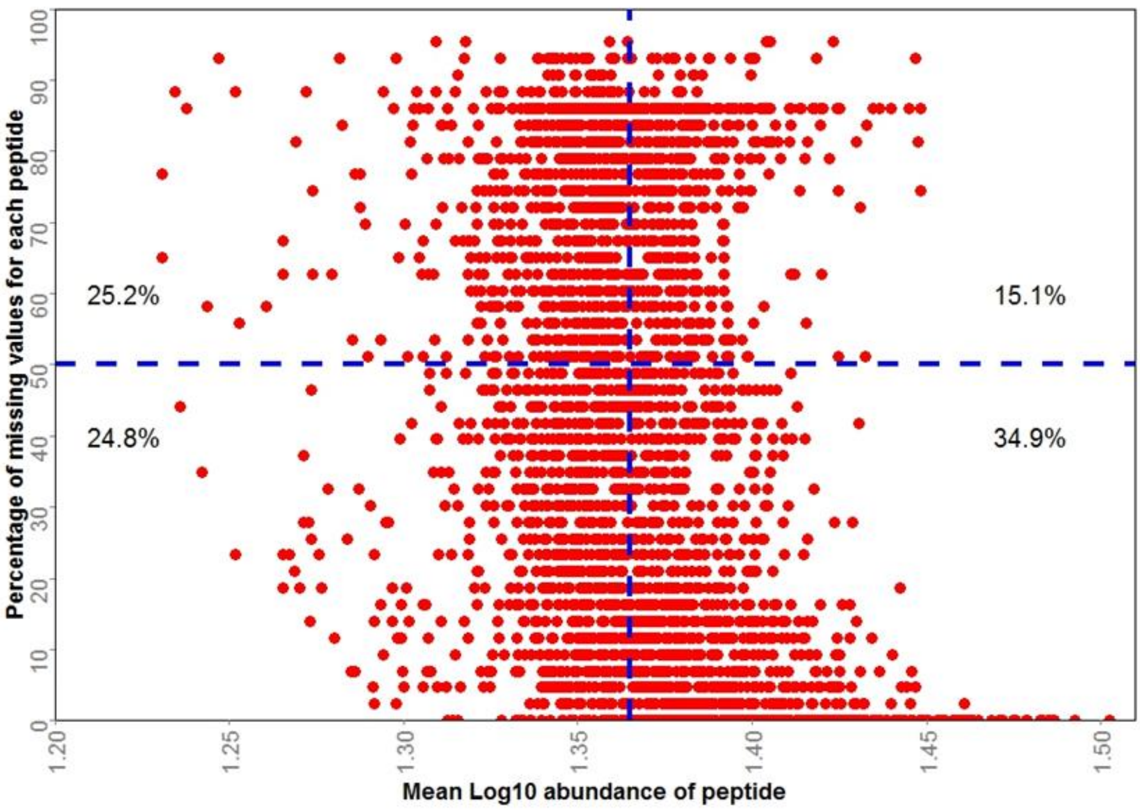

Supplementary Figure S1. Correlation between log_{10} abundance and percentage of missing values in each peptide. Vertical and horizontal dashed lines represent the median abundance and 50% of missing values, respectively. Correlation value is -0.31.

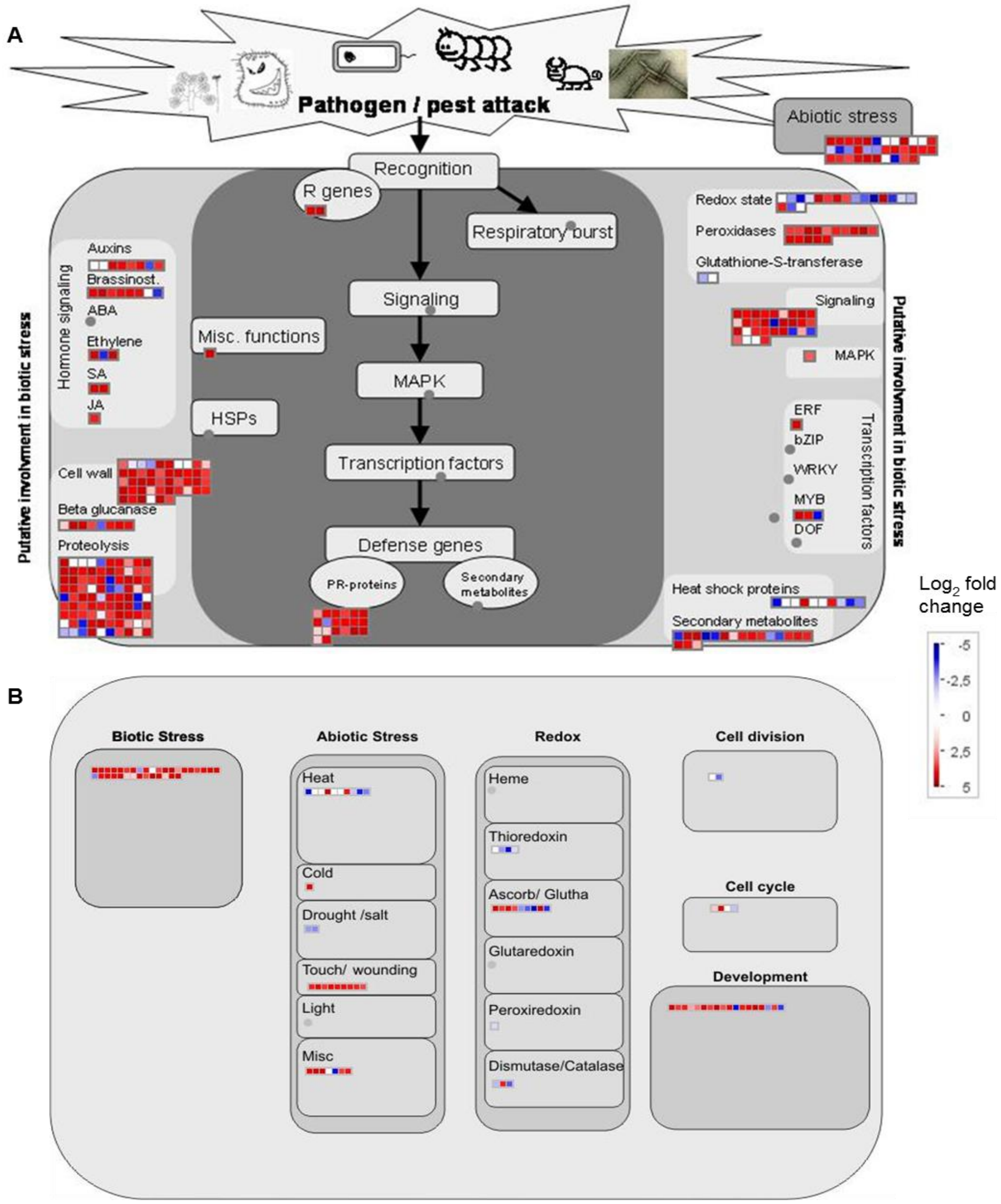

Supplementary Figure 2. Biotic and abiotic stress response overview in field- and greenhouse-grown potato inferred from the Mapman pathway analysis of differentially abundant apoplastic proteins with false discovery rate < 0.001 (according to the Benjamini−Hochberg procedure for determining q). Mapman pathways for biotic and abiotic stress response overview (A) and for cellular response overview (B). Red represents proteins with increased abundance in greenhouse samples compared to that in field samples. ABA (abscisic acid), SA (salicylic acid), JA (jasmonic acid), HSPs (heat shock proteins), resistance genes (*R*-genes), MAPK (Mitogen-activated protein kinase), PR-proteins (pathogenesis related proteins), bZip (Basic Leucine Zipper Domain-transcription factors), WRKY (WRKY domain--transcription factors), MYB (MYB domain-transcription factors), Dof (DNA-binding with one finger).
